# A continuous ideal free distribution approach to the dynamics of selfish, cooperative and kleptoparasitic populations

**DOI:** 10.1098/rsos.160788

**Published:** 2016-11-30

**Authors:** Ilona Reding, Michael Kelley, Jonathan T. Rowell, Jan Rychtář

**Affiliations:** 1 University of North Carolina at Wilmington, Wilmington, NC, USA; 2 Appalachian State University, Boone, NC, USA; 3 University of North Carolina at Greensboro, Greensboro, NC, USA

**Keywords:** ideal free movement, sympatry, cooperation, interference competition, spatial structure, kleptoparasitism

## Abstract

Population distributions depend upon the aggregate behavioural responses of individuals to a range of environmental factors. We extend a model of ideally motivated populations to describe the local and regional consequences of interactions between three populations distinguished by their levels of cooperation and exploitation. Inspired by the classic prisoner's dilemma game, stereotypical fitness functions describe a baseline non-cooperative population whose *per capita* fitness decreases with density, obligate co-operators who initially benefit from the presence of conspecifics, and kleptoparasites who require heterospecifics to extract resources from the environment. We examine these populations in multiple combinations, determine where both local and regional coexistence is permitted, and investigate conditions under which one population will invade another. When they invade co-operators in resource-rich areas, kleptoparasites initiate a dynamic instability that leads to the loss of both populations; however, selfish hosts, who can persist at low densities, are immune to this risk. Furthermore, adaptive movement may delay the onset of instability as dispersal relieves dynamic stress. Selfish and cooperative populations default to mutual exclusion, but asymmetric variations in interference strength may relax this condition and permit limited sympatry within the environment. Distinct sub-communities characterize the overall spatial structure.

## 1. Introduction

Competition, cooperation and other interactions influence the persistence or exclusion of populations within a community. Individual responses to these factors—and others such as resource availability, environmental quality and risk of predation—shape distributions over regional scales [1]. Competition's role in setting ranges is increasingly understood [2–6], but our theoretical knowledge of the role that cooperation, parasitism and intra-guild predation play is just beginning (e.g. [7]). Parasitism, for example, affects nearly all species, and empirically it influences the structuring of communities [8–10], yet corresponding mathematical studies remain sparse. In counterpoint, cooperation and its vulnerability to exploitation by cheaters has inspired considerable work by game theorists using various extensions of the classic prisoner's dilemma (e.g. [11–13]), but the resulting models often lack a biological foundation. In this paper, we employ a modelling framework that bridges game theory and ecological modelling to study the pairwise and collective interactions of three populations characterized by distinct levels of cooperation or parasitism. We examine both the local population dynamics and the spatial arrangements that arise when individuals adaptively disperse across the landscape.

Consumers of common resources primarily interact via competition through territoriality, interference and resource depletion. The frequency of competitive encounters varies from solitary animals that maintain relatively large territorial ranges [14–16] to groups of more social species which may conflict while patrolling borders [17]. Close proximity within-group can also be a source of interference and produce physical barriers to movement, increase the incidence of conspecific attacks or degrade habitat quality. Illustrating that last possibility, roe deer (*Capreolus capreolus*) trample patches during highly concentrated foraging during winter [18]. Even when there are secondary benefits for associating (herd protection, predator alarms, etc.), individuals largely survive by their own efforts.

Other social species do demonstrate cooperation in their actions. Eusociality in insects is the most recognized example of animal cooperation in which individuals sacrifice their own reproductive potential to aid conspecifics or raise the offspring of others [19]. Red harvester ants, *Pogonomyrex barbatus*, employ an age-dependent division of labour with young workers engaged in brood care and older workers foraging outside of the nest [20]. Individual survival is often low for obligate co-operators [21], and a minimum group size is required for successful activity. Common predatory pack animals improve hunting efficiency and can bring down larger prey by increasing pack size, while African wild dogs, *Lycaon pictus*, are also obligate cooperative breeders that need conspecific helpers for foraging, breeding, and deterring natural enemies [22]. Dolphins and killer whales are known to herd fish into bait balls through carouseling [23], while humpbacks utilize bubble netting to similar effect with krill [24]. Such activities also allow other predators (e.g. swordfish, sharks and gulls) to rush in and claim a share of the prey. Cooperative benefits are not limitless, however, and continued increases to group size or labour division may negatively affect the overall health of the population as individuals' shares of resources decline.

A number of possible mechanisms supporting the promotion of cooperation have emerged from game theoretic studies. These include kin selection [25], partner selection [26,11], role-model selection [12], group size, age structure and memory [27–29], social diversity and variation of the strength of cooperation [30], and tit-for-tat strategies [31]. See Wang *et al.* [13] and citations therein for an extensive review of game theoretic treatments. Experimental results also point to spatial structure and expansion waves as mechanisms maintaining cooperation [32].

Contra cooperation, kleptoparasitism occurs when one animal surreptitiously or aggressively steals resources (e.g. food, nesting material) gathered by another rather than obtaining them independently [33]. Kleptoparasitism is a common phenomenon throughout the taxa [8,34,35]. Examples can be found among birds (frigate-birds, *Fregata* spp., and skuas, *Stercorarius* spp. [36]), hymenopterans like Spinola bees, *Radoszkowskiana rufiventris* [37], and the wasp *Argochrysis armilla* [38], spiders like *Argyrodes elevatus* [39], fish (the Western Buffalo bream, *Kyphosus cornelii* [40]) and mammals (the spotted hyena, *Crocuta crocuta*) [41], and even budding yeast where one strain can catalyse the conversion of sucrose and another cannot [32]. Some kleptoparasites force their host-victims to share items or drive them away completely ([42], and citations therein). Hyenas and lions claim kills taken by others who cannot successfully defend their spoils against interlopers. Similarly, Arctic skuas intercept auks returning to their nests with fish [33], and the gecko *Phelsuma inexpectata* steals bee pollen [43]. Less confrontational, fork-tailed drongos use false alarm calls to parasitize both birds and small mammals ([44]; modelled by [45]), a tactic also used by shrike tanagers in aerial tumbles [46], while red-faced spinetails steal untended nest material [35]. Even brood parasitism is a variation on this general concept, notable in the Apidae bee family [9,47] and cowbirds. The shiny cowbird (*Molothrus bonariensis*) has endangered the yellow-shouldered blackbird (*Agelaius xanthomus*) in Puerto Rico since 1976 [48], and in general brood parasitism threatens several species of birds [49].

In this paper, we extend a reaction–advection model of ideally motivated populations [2,3] to describe interactions between three stereotypical populations consisting of selfish individuals, co-operators and kleptoparasites. We consider these populations in isolation and pairwise interactions before concluding with an examination of the dynamics when all three are present. Through a combination of analytic and simulation approaches, we detail the conditions under which one group can invade another and where persistent local and regional coexistence is possible. Kleptoparasites may invade any host population of sufficient size, which is functionally invariant across host types. Where invasion is possible, kleptoparasites and selfish hosts always reach stable coexistence; however, stability becomes resource-dependent with cooperative hosts. High resources excite oscillations in population levels that eventually depress host density below a sustainability threshold, culminating in the loss of both populations; however, adaptive movement may delay the onset of instabilities. Selfish and cooperative populations are intrinsically mutually exclusive at the local level. Asymmetries in interference strengths can relax this situation and permit limited local sympatry (cf. [3]), but increasing resources will reestablish exclusion. Finally, regional communities are spatially structured, with local compositions stratified by resource values.

## 2. Model

This section extends a reaction–dispersal model for ideally motivated populations [2] to three interacting populations distinguished by varying degrees of cooperation and exploitation. Ideally motivated individuals migrate in the direction of greatest immediate increase in fitness in a continuous analogue to the ideal free distribution ([50]; also see [51]). Only some portions of total fitness are directly measurable by individuals, e.g. resource availability and competition levels, while others, such as mortality risks, may be obscured. Imperfect knowledge of the spatial variation in those unobservable factors leads to source-sink dynamics ([2]; J.T.R. 2016, unpublished manuscript); however, this paper will not explore that aspect of the model.

Consider a population with local density *u*_*i*_(**x**,*t*) at position **x** and time *t* that is subject to an observable fitness function *f*_*i*_(*R*,*u*_*i*_,*). This function represents the *per capita* amount of resources extracted from the environment based upon local resource availability *R*(**x**), conspecific density *u*_*i*_, plus other factors including heterospecifics. Gathered resources are converted to reproductive growth with metabolic efficiency *r*_*i*_, and the population suffers a uniform *per capita* mortality rate *μ*_*i*_. These last two processes are non-observable and do not affect movement. Density flows in the direction of improved fitness and results in a net change due to immigration equal to −*k*_*i*_∇⋅(*u*_*i*_∇*f*_*i*_), where *k*_*i*_ is the sensitivity to local changes in the fitness landscape. The complete equation of change for population density is thus

2.1 $$\frac{\partial u_{i}}{\partial t}=-k_{i}\nabla \cdot (u_{i}\nabla f_{i})+r_{i}u_{i}\left(f_{i}-\frac{\mu_{i}}{r_{i}}\right).$$

In a local, non-spatial model, the corresponding system of ordinary differential equations (ODEs) would be

2.2 $$\frac{du_{i}}{dt}=r_{i}u_{i}\left(f_{i}-\frac{\mu_{i}}{r_{i}}\right).$$

### 2.1 Dynamic and spatial equilibrium

**Figure 1.**
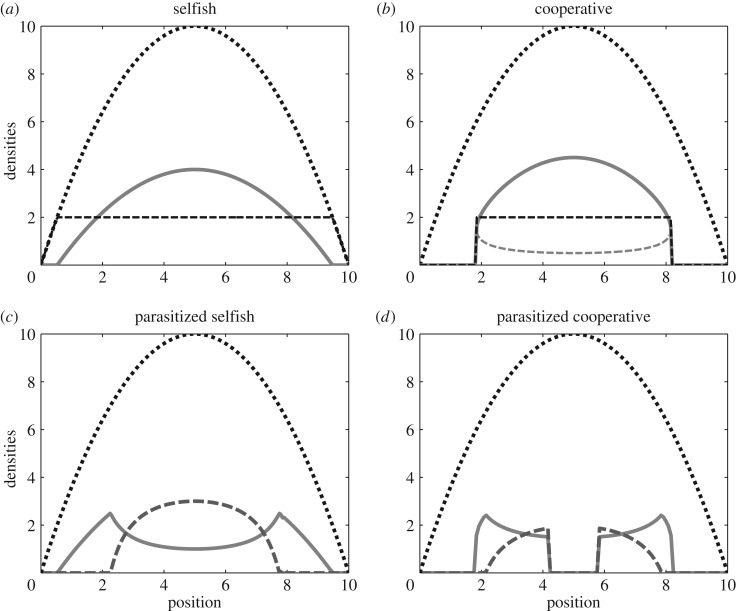
Isolated and parasitized hosts. Isolated host populations have long-term distributions (solid grey) that are at dynamic equilibrium and are ideal with uniform fitness (dashed) for a given resource curve (dotted). (*a*) Selfish population in isolation. (*b*) Cooperative population in isolation (unstable lower solution also shown). (*c*) Introduction of kleptoparasites (dashed grey) to selfish hosts results in new ideal distribution at steady state. (*d*) Parasitism risks destabilizing the central area of cooperative populations with excessive resources. For all panels, host parameters are *μ*_*i*_=2, *r*_*i*_=1, *a*_*ij*_=1, *h*=1 and *α*=1.5. Parasitic parameters are *μ*_3_=1, *r*_3_=1 and *θ*_*i*_=0.205. Sensitivity to fitness is a common value *k*=0.001. Resources are *R*(**x**)=10−0.4(**x**−5)^2^.

Per Rowell [2], a rapid or highly motile population described by model equation (2.1) approaches a distribution wherein fitness has a uniform value *f*_*i*_=*E*_*i*_ wherever *u*_*i*_>0 and *f*_*i*_≤*E*_*i*_ at locations immediately neighbouring the inhabited region (figure 1*a*,*b*). Even when multiple species are admitted regionally, the populations generate jointly ideal distributions [3]. Local dynamic equilibrium is achieved either when *u*_*i*_=0 or *f*_*i*_=*μ*_*i*_/*r*_*i*_. The latter is also consistent with balanced migration for ideally motivated competitors.

### 2.2 Selfishness, cooperation and exploitation

The communities under consideration are composed of two or three populations whose fitness functions are stereotypical expressions of selfishness, cooperation or exploitation (*i*=1,2 and 3, respectively),

2.3a $$\begin{array}{rl} f_{1} & =(1-\theta_{1}u_{3}){\tilde{f}}_{1}(u_{1},u_{2})=(1-\theta_{1}u_{3})\frac{R}{a_{11}u_{1}+a_{12}u_{2}^{2}+h_{1}} \end{array}$$

2.3b $$\begin{array}{rl} f_{2} & =(1-\theta_{2}u_{3}){\tilde{f}}_{2}(u_{1},u_{2})=(1-\theta_{2}u_{3})\frac{Ru_{2}}{a_{21}u_{1}+a_{22}u_{2}^{2}+\alpha_{2}^{2}}, \end{array}$$

2.3c $$\begin{array}{rl} \text{and}\;\;f_{3} & =\theta_{1}u_{1}{\tilde{f}}_{1}+\theta_{2}u_{2}{\tilde{f}}_{2}. \end{array}$$

Rowell [2] previously described the first two host populations in isolation (*u*_*j*_=0,*j*≠*i*). Direct competition between the two hosts is a novel development, as is the introduction of the exploitative functional type to this model framework (*i*=3). The host functions derive from first and second degree saturation curves describing total grazing efforts with interference interpreted as prolonging resource retrieval time. *h*_1_ and $\alpha_{2}^{2}$ correspond to intrinsic resource collection times. In this model, a scramble kleptoparasitism transfers a portion *θ*_*i*_ of gathered resources upon encounters with host-type individuals (e.g. via false alarm calls, combative threat or surreptitious theft).

The population of ‘selfish’ or ‘self-reliant’ individuals (*i*=1) has been studied with regard to range limits [2], performance trade-offs [3] and harvesting (J.T.R. 2016, unpublished manuscript). Increased density reduces individual fitness by prolonging the time required to obtain local resources due to interference competition (equation (2.3*a*)), ∂*f*_1_/∂*u*_1_<0. These populations are excellent pioneer species and disperse across heterogeneous landscapes to every contiguous location where resources sustain a local population, *R*>*μ*_1_*h*_1_/*r*_1_. Previous theoretical results, heuristic arguments and numerical simulations strongly supported the conclusion that the uniformly-fit distribution

2.4 $$u_{1}^{*}(\text{x})=\frac{1}{a_{11}}\left(\frac{R(\text{x})}{E_{1}^{*}}-h_{1}\right)$$

is the global attractor for the population under (2.1). Here, *a*_11_ is the selfish intraspecific interference strength and E*_1_=*μ*_1_/*r*_1_ is the ratio of mortality to metabolic efficiency.

By contrast, ‘contributors’ (*u*_2_) co-operate to increase personal fitness by more effectively gathering resources, hunting larger prey or sharing nest-site responsibilities. The corresponding fitness function features an Allee effect (2.3*b*), and initially increases with density before declining as resources become exhausted. This function generates a strong tendency towards aggregation into clusters, which can result in spatial instabilities and a potential absence of well-posedness in the model similar to chemotaxis. Unlike that phenomenon, however, self-regulation in the present model prohibits unbounded growth. This instability is ecologically meaningful as it indicates mosaics or patchy spatial distribution across the landscape. For any uniform fitness value *E*_2_ (with the dynamic equilibrium value E*_2_=*μ*_2_/*r*_2_), equation (2.1) admits two principal ideal distributions (figure 1*b*)

2.5 $$u_{2}(\text{x})=\frac{1}{2a_{22}E_{2}}\left(R(\text{x})\pm \sqrt{R^{2}(\text{x})-4a_{22}E_{2}^{2}\alpha_{2}^{2}}\right).$$

In practice, a movement-balanced distribution can be patchy and draw from both ideal curves and the trivial solution. The unstable lower positive solution for $E_{2}^{*}$ (denoted ${\hat{u}}_{2}^{*}$) constitutes a minimum threshold for local persistence in the ODE problem (2.2). This threshold decreases as resources improve, but because of reliance upon conspecifics, contributors are poor colonizers both locally and regionally.

The final population type (*u*_3_) represents an obligate kleptoparasite that steals resources gathered by either of the previous two populations. This kleptoparasite cannot independently retrieve resources from the environment and is entirely dependent upon the presence of heterospecifics for survival. A fraction of resources ($\theta_{i}{\tilde{f}}_{i}$) are transferred upon each encounter with heterospecifics, which occurs under simple mass action encounter rates (*u*_3_*u*_*j*_). The parasitized host's fitness (2.3*a,b*) is commensurately reduced by a factor of (1−*θ*_*j*_*u*_3_).

## 3. Results

### 3.1 Kleptoparasitic invasions

Kleptoparasites persist only in the presence of heterospecifics engaged in resource recovery; therefore, they are placed in the role of invader in any pairwise interaction (summarized in table 1). We assume that a host population has already established its intrinsic ideal equilibrium, $u_{i}^{*}$. For cooperative hosts, which exhibit two solutions for any given fitness level, the locally attracting upper solution will be chosen. Parasite density depresses host fitness through the transfer of resources (∂*f*_*i*_/∂*u*_3_<0); however, conspecific density has no direct effect on the parasites' own fitness (∂*f*_3_/∂*u*_3_=0). Self-regulation is an indirect consequence of the reduction in host density.

**Table 1.** Exploiter invasion. Host properties when kleptoparasites invade, including host density at coexistence and the resources levels necessary for host persistence, invasibility and destabilization of coexistence.

host	selfish	cooperative
host density:	$\frac{h}{\theta_{1}R(r_{3}/\mu_{3})-a_{11}}$	$\frac{\alpha }{\sqrt{\theta_{2}R(r_{3}/\mu_{3})-a_{22}}}$
host-viable res.:	h1(μ1r1)∫	$2\alpha \left(\frac{\mu_{2}}{r_{2}}\right)\sqrt{a_{22}}$
invasion res.:	(a11θ1)(μ3r3)+h1(μ1r1)∫	$\left(\frac{a_{22}}{\theta_{2}}\right)\left(\frac{\mu_{3}}{r_{3}}\right)+\theta_{2}\alpha^{2}{\left(\frac{\mu_{2}}{r_{2}}\right)}^{2}\left(\frac{r_{3}}{\mu_{3}}\right)$
instability res.:	n.a.	2a22θ2(μ3r3)∫

Two invasion features are consistent across hosts. First, there is a minimum host density required to promote an initial incursion. Host self-regulation also downregulates parasites. When parasite density is initially small (*u*_3_≈0), the host fitness equals E*_i_=*μ*_*i*_/*r*_*i*_, and the parasite invades if the isolated host density exceeds a threshold set by the relative equilibrium fitness levels (*μ*_*i*_/*r*_*i*_) and the transfer rate *θ*_*i*_,

3.1 $$u_{i}^{*}>\left(\frac{1}{\theta_{i}}\right)\left(\frac{\mu_{3}}{r_{3}}\right)\left(\frac{r_{i}}{\mu_{i}}\right).$$

Second, when coexistence occurs at the non-trivial equilibrium $\left({\tilde{u}}_{i},{\tilde{u}}_{3}\right)$, the parasite equilibrium density is defined relative to host density by a simple linear equation

3.2 $${\tilde{u}}_{3}=\frac{1}{\theta }-{\tilde{u}}_{i}\frac{\left(\mu_{i}/r_{i}\right)}{\left(\mu_{3}/r_{3}\right)}.$$

The appendix A details the derivation of these general results.

If the host population consists of selfish individuals (*i*=1), the minimum density condition (3.1) may be equivalently restated as a threshold condition on the local resource levels

**Figure 2.**
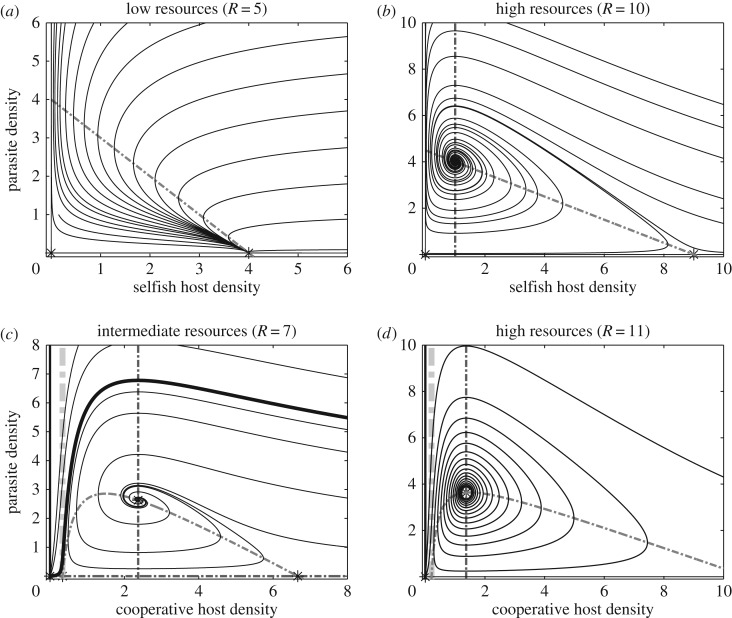
Parasite–host dynamics. Each panel shows the phase plane of a local (non-spatial) two-population model under different resources. Nullclines (broken lines) are shown for both host (lighter grey) and parasite (darker grey). (*a*) A marginal value refuge for the selfish host in which the parasite cannot persist. (*b*) Parasites persist at higher resource levels with selfish population. (*c*) Parasitism of co-operators divides the phase plane into two basins of attraction for (0,0) and coexistence state (${\tilde{u}}_{2},{\tilde{u}}_{3}$). (*d*) Parasitism destabilizes the system at high resources, and both populations are lost. All parameters as in figure 1.

**Figure 3.**
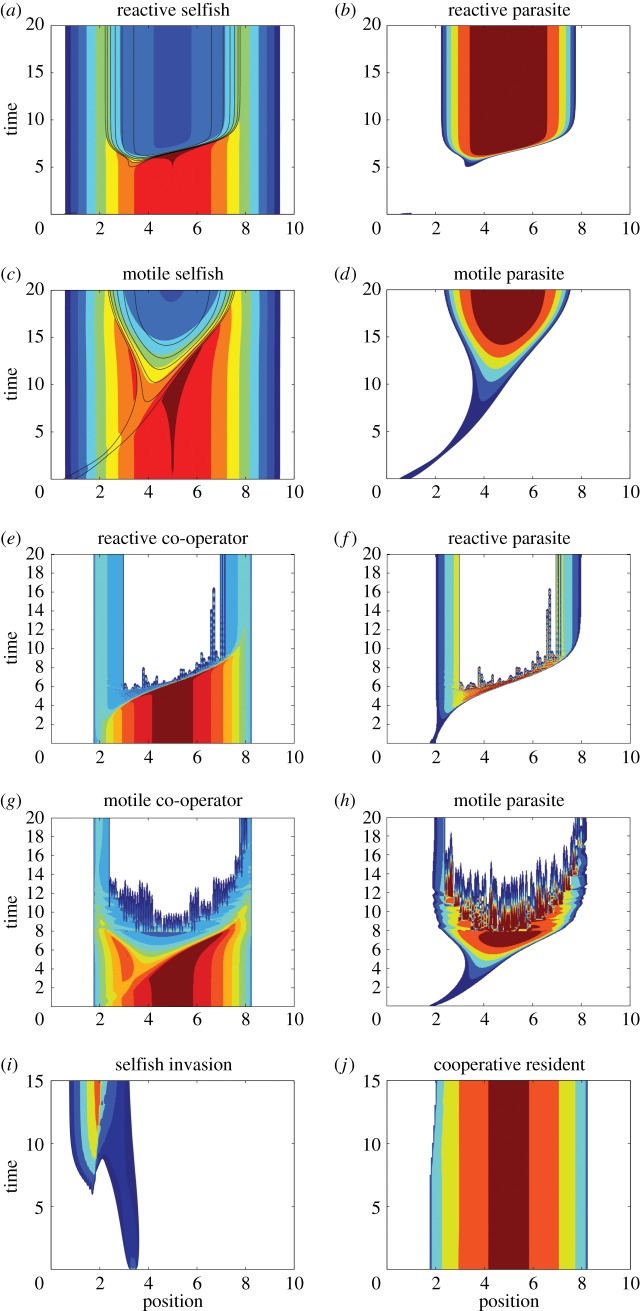
Spatial structure of pairwise interactions. For each pair of species (by row), an invader population is introduced on the left of the inhabited environment at small number. Reactive populations perform local dynamics more quickly than movement with *r*_*i*_=10, *μ*_1_=*μ*_2_=20 and *μ*_3_=10 while *k*=0.1. Motile populations have parameters as in figure 1, but *k*=1. Heat maps: white indicates population density below detection threshold (0.1), while dark red represents 5+/2.5+ densities for hosts/parasites. During response of reactive selfish hosts (*a*) to spread of reactive parasites (*b*), displacement is similar to travelling wave. By contrast, motile parasites centralize (*d*) before expanding outward within the host's range (*c*). Level curves for parasite density are overlayed atop host heat maps. Reactive co-operators retain marginal and adjacent territories (*e*) as parasites create a disruption wave that isolates the community (*f*). In mobile communities (*g*,*h*), the disruption only occurs after the parasite is widespread. In host competition under weak interspecific interference (*a*_12_=0.2 and *a*_21_=0.5), selfish invaders manifest two populated regions that eventually merge (*i*). The resident co-operators are largely unaffected (*j*). Resources in (*e*–*j*) are *R*(*x*)= 11.25−0.5*x*^2^.

3.3 $$R>R_{\mathrm{inv}}=\left(\frac{a_{11}}{\theta_{1}}\right)\left(\frac{\mu_{3}}{r_{3}}\right)+h_{1}\left(\frac{\mu_{1}}{r_{1}}\right),$$

which surpasses that required to sustain the host, *R*_via_=*h*_1_*μ*_1_/*r*_1_. These resource levels delimit regions of the environment supporting one of three possible outcomes for local dynamics. In areas of extremely low resources (*R*≤*R*_via_) neither population is present. At intermediate resource levels (*R*_via_<*R*≤*R*_inv_), the selfish host persists alone as its density is insufficient to support kleptoparasitism (figure 2*a*). In regions of abundant resources (*R*>*R*_inv_), parasitized coexistence is sustainable (figure 2*b*). When adaptive movement is incorporated into the model, the colonizing capacity of selfish individuals ensures that the hosts retain their full natural range, with the area of coexistence centralized about resource peaks and a host refuge in resource-limited areas. At the jointly ideal equilibrium distribution, resource peaks coincide with peaks in parasite density, while the host distribution exhibits a conspicuous depression in these areas (figure 1*c*). Numerical simulations (see appendix Ae), such as those shown in figure 3*a*–*d* and 2*a*,*b*, and the absence of additional local attractors from which to construct a spatial distribution strongly support ideal regional coexistence as a global attractor. The relative speed of movement shapes the evolution of the community. In relatively motile communities (*k*≥*r*, *μ*), invading kleptoparasite centralizes before expanding and displacing the host (figure 3*c*,*d*). If the community is more reactive (*r*, *μ*>*k*), the invasion resembles a travelling wave (figure 3*a*,*b*). Moreover, the adaptive movement dampens or eliminates the spiralling observed in the non-spatial local dynamics.

The invasion condition (3.1) remains identical when the host population is composed of co-operators; however, the region vulnerable to invasion is set by different resource levels (table 1), which may or may not exceed that required for a viable host population. The most marginalized co-operator population boasts a density $u_{2}^{*}=\alpha_{2}/\sqrt{a_{22}}$, a level which is remarkably independent of the metabolic efficiency and mortality of the host. The loss of a refuge for co-operators occurs when the theft parameter exceeds

3.4 $$\theta_{2}\geq \frac{\sqrt{a_{22}}}{\alpha_{2}}\left(\frac{\mu_{3}}{r_{3}}\right)\left(\frac{r_{2}}{\mu_{2}}\right).$$

Typically, the resource value at which parasitic invasions occur exceeds the host-viability level; however, when (3.4) holds, the lower-resource refuges do not exist, and the local host population is everywhere at risk of non-recoverable destabilization (see below).

The most important difference between hosts is that host–parasite coexistence is locally stable only under intermediate resources for cooperative hosts. At particularly high resources, *R*>2(*a*_22_/*θ*_2_)(*μ*_3_/*r*_3_), local coexistence destabilizes, and the population trajectory spirals out until it crosses the minimum population threshold necessary for co-operators to persist (figure 2*d*), after which both the host and parasite irrevocably decline and are lost. The transient phase characterized by the expanding population spirals can be of considerable duration.

In the spatial model, the destabilization at high resources and the population's natural inclination toward clustering render large swathes of in principle viable habitat vacant in an ideal community (figure 1*d*). The unstable local dynamics may couple with adaptive movement (and its potential for spatial instability) to produce patchy distributions that only broadly match the ideal distribution (figure 3*e*–*h*). In more motile communities, the initiation of excitement is delayed, as movement relieves dynamic pressures and maintains a near-ideal community distribution until central hosts are pushed towards the persistence threshold.

### 3.2 Host exclusion

**Figure 4.**
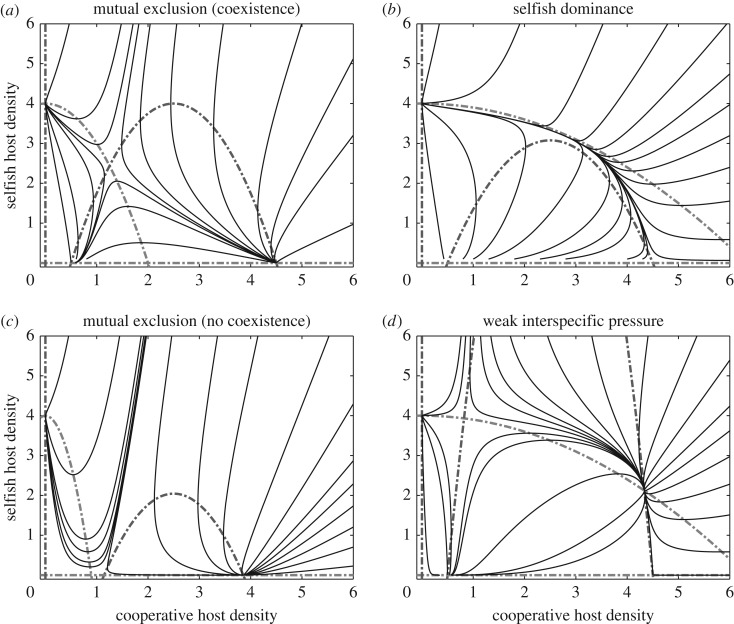
Local host interactions. Selfish and cooperative populations compete locally under different interference strengths. Nullclines are shown for selfish (light grey) and cooperative populations (dark grey), along with trajectories. (*a*) Coexistence state is a saddle (*a*_*ij*_=1). (*b*) Selfish dominance (*a*_21_=1.3, *a*_12_=0.1). (*c*) Mutual exclusion without coexistence (*α*=2.1, *a*_12_=5, *a*_21_=0.9). (*d*) Weak interspecific interference adds a second, stable coexistence state (*a*_12_=0.1, *a*_21_=0.3).

Local competition between selfish and cooperative populations leads to one of four distinct dynamic scenarios. Figure 4 provides representative phase portraits for each case. The non-trivial nullcline of each population is parabolic, one symmetric about *u*_2_=0 (selfish) and the other symmetric about *u*_2_=*Rr*_2_/(2*a*_22_*μ*_2_) (cooperative). The primary outcome features mutual exclusion of the hosts with coexistence only at a saddle. This results when interference strengths are balanced (*a*_11_*a*_22_=*a*_12_*a*_21_) and the co-operators' upper solution is resistant to invasion. The three remaining cases are strict dominance by self-reliant individuals, mutual exclusion where the saddle occurs at the minimally viable semi-trivial solution $\left({\hat{u}}_{2}^{*},0\right)$, and a dynamic that admits two distinct coexistence equilibria. In this last scenario, asymmetric exclusion favours the selfish population, which still cannot be invaded, while the second coexistence state is a local attractor when co-operators are more established. In general, selfish hosts may invade established co-operators when

3.5 $$R>\frac{\mu_{1}}{r_{1}}(h+a_{12}{u_{2}^{*}}^{2}).$$

These last three cases are tenuously modulated by variations in intra- and inter-specific interference which influence the width and/or height of the nullclines. As resource levels increase, the dynamics always transition to the mutual exclusion of the two host populations. This development rests largely on changes to the minimum viable co-operator population—which goes towards 0—and the maximum co-operator population, whose growth outpaces the expansion of the selfish population's nullcline (*R* versus $\sqrt{R}$). Because of this dependence on resources and interference strengths, different locations may exhibit distinct dynamic behaviours, with local dominance, coexistence and mutual exclusion all present. Figure 3*i*,*j* illustrates the community's spatial structure under a selfish invasion of established co-operators with weak inter-specific interference. Although marginalized, selfish invaders establish their own exclusive territory and co-opt or share neighbouring co-operators' territory. The resident hosts maintain a firewall at higher resources, so the selfish group cannot populate the far end of the environment.

### 3.3 Community dynamics

**Figure 5.**
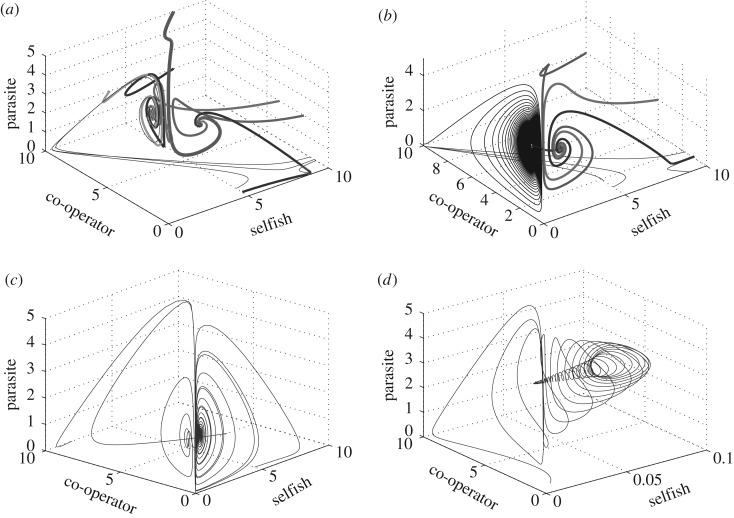
Community evolution: different trajectories for the three-species community dynamics are shown when the hosts remain mutually exclusive. (*a*) *θ*_*i*_= 0.15, two local host–parasite attractors. (*b*) Co-operators persist against parasites, but are weakened sufficiently for selfish hosts to invade (*θ*_*i*_=0.20). (*c*) Co-operators are destabilized in the face of parasitism (*θ*_*i*_=0.30). (*d*) In-depth look at local behaviour of co-operator–parasite equilibrium where host is weakened but not yet vulnerable to invasion by selfish hosts (*θ*_*i*_=0.19). In all plots, resources are set to *R*=10, while all other parameters equal 1.

In a local, non-spatial model of community dynamics, kleptoparasitism introduces oscillatory or spiralling behaviour in the evolution of host–parasite levels. Under normalized interference (*a*_*ij*_=1), host exclusion is a characteristic but not persistent feature of the local dynamics. The presence of kleptoparasites depresses the densities of both hosts by creating an apparent reduction in resource values. This drop in host density may be sufficient to guarantee selfish dominance or at least selfish promotion. For low levels of kleptoparasitism, the hosts maintain their mutual exclusion and there are two locally stable semi-trivial solutions featuring the parasite and one of the host types (figure 5*a*). The Allee effect ensures $\left(u_{1}^{*},0,u_{3}^{*}\right)$ remains locally stable, but should parasitism result in the condition

3.6 $$\theta_{1}u_{3}^{*}<1-\frac{1}{R}\frac{\mu_{1}}{r_{1}}(h+a_{12}{u_{2}^{*}}^{2}),$$

then we arrive at an interesting scenario where the cooperative host–parasite equilibrium can remain locally stable under the restriction *u*_1_=0, but it is unstable within the community dynamics as selfish hosts can invade (figure 5*b*). There is no corresponding analogous behaviour with the semitrivial solution $\left(u_{1}^{*},0,u_{3}^{*}\right)$ because of the Allee effect imposed on co-operators.

**Figure 6.**
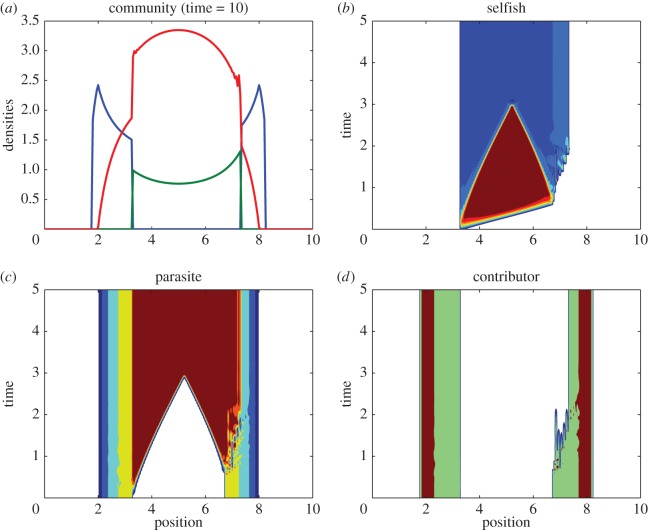
Sequential introductions. A small selfish population is introduced to the community already containing co-operative and exploitative populations. (*a*) Community composition at end of simulation is almost ideal but not symmetric. Red, parasite; blue, co-operator; green, selfish. (*b*) Selfish invaders move rapidly across the uninhabited interior. (*c*) Parasites trail selfish hosts at reduced speed. (*d*) Co-ooperators on left are mostly unaffected by the community change. Those on the right are disrupted upon contact with the selfish expansion front. All parameters as in figure 3 for reactive populations, and *R*(*x*)=11.25−0.5*x*^2^.

Cooperative clustering limits commentary about the spatial model's global asymptotic behaviour, but we can broadly speak of regions where general phenomena are likely to be encountered. For illustrative purposes (figure 6), consider a scenario of sequential introductions: first co-operators, then parasites and finally selfish individuals. The community reaches an ideal steady state distribution before each new population is introduced at low densities. Upon the arrival of selfish individuals, the community resembles figure 1*d*, and they perceive two relatively high-valued areas ( *f*_1_) that could attract colonization. First, selfish individuals can monopolize untapped marginal resources at the periphery of the co-operators' range. The second potential aggregation site centres around the resource peak, which may or may not be vacated as a consequence of the preceding kleptoparasitic invasion. The co-operators' parasite-free zone isolates these areas during migration. If selfish indviduals arrive at the outer margins, they persist there with little change to the rest of the community (not pictured). Should they instead appear within the parasitized sub-population (e.g. a random transplant or mutation event), the depression in co-operator density provides a clear migratory path to the vacant centre (figure 6*b*). The parasite responds to this new prey slowly (figure 6*c*). The selfish expansion wave also disrupts resident co-operators in the distant sub-group upon contact, but they are not lost (figure 6*d*). The community nears an ideal joint distribution at the end of the simulation, (figure 6*a*).

In general, the community will evolve towards a constellation of distinct sub-communities. In order of increasing resources, possible areas are described as uninhabited, selfish-only, co-operator-only, parasitized co-operators and finally parasitized selfish individuals. The existence and precise width of each of these respective zones depends upon the various model parameters as well as the initial distributions of populations and the potential spatial instabilities of motivated co-operators. As in the previous section, variation of the interference strength *a*_*ij*_ further opens the possibility of host coexistence. The dynamic behaviour of the two host populations remains resource-mediated, so alternative spatial distributions, potentially including coexistence of hosts or all three populations, can be generated in different areas of the environment (not pictured).

## 4. Discussion

This paper extends a model of ideally motivated populations [2] to niche or guild competitors with fundamental functional differences in the fitness of their respective members. We elected to use three distinct fitness functions that were ecologically meaningful and captured features of the prisoner's dilemma from game theory. Per the name, co-operators benefited from the presence of conspecifics through an Allee effect, while selfish individuals and kleptoparasites were alternate versions of the ‘Defect’ strategy. At low densities, co-operators are ineffective in both resource recovery and deterrence of niche competitors, and the relationship to selfish rivals was more akin to an ammensal threat. Kleptoparasites represented a clear case of exploitation. These obligate parasites could not extract native resources on their own and negatively affected host fitness, but conspecifics imposed no direct reduction on the parasites' own fitness. Fitness loss came indirectly as host levels were depressed (see grazing model in [2]). A spatial structure for the regional community naturally emerged from the combination of adaptive movement and local dynamics.

We established that a minimum host density was necessary to promote parasitic invasions, yet this did not necessarily guarantee persistence of the parasite. Instabilities developed during invasions of cooperative hosts in resource-rich environs because the subsequent bloom in parasites would be so great as to depress host levels below the point where they could recover even if the invaders were removed. A parasitized host's density declined relative to resource richness. Although the transfer of resources was observable to both parties, the parasite tracked total resource uptake of the host population, $\theta_{i}u_{i}{\tilde{f}}_{i}$, while the host sought to optimize individual fitness subject to total parasitism, $(1-\theta_{i}u_{3}){\tilde{f}}_{i}$. This discrepancy in perceptions creates an apparent mismatch at each link of the tri-trophic food chain. Most hosts vacate resource peaks because of the loss from extreme theft, but any remaining individuals are incredibly productive and support a disproportionately large contingent of kleptoparasites.

It was previously known that selfish populations easily dispersed, while cooperative populations were natural aggregators [2]. The two host populations are principally exclusive of one another at the local scale because their relative contributions to interference pressures is volatile. Selfish individuals exerted greater interference when both densities were low while co-operators synergistically dominated at high densities as clustering formed mutually supporting networks. Building up the number of co-operators in a self-reinforcing group has been a documented mechanism to promote cooperation amid structured networks [11,27]. Although this is a direct consequence of our choice of fitness functions, it reasonably reflects the organizational principle underlying cooperation. Any alternative model that transitioned co-operators from a small, disorganized group to a large well-organized mass should behave similarly.

Asymmetric variation in interference strengths mediated exclusion (figures 3*i*,*j* and 4). Reduced interspecific interference supports stable local coexistence and limited zones of sympatry in the spatial model. The host types are not equally invadable. Selfish individuals can establish themselves amid co-operators, but the reverse does not hold because of the minimum density required for persistence. At the regional scale, cooperative clustering might recruit members quickly enough to amass local density sufficient to overcome this dynamic limitation. Even with favourable parametric conditions, however, local dynamics inevitably return to a state of exclusion as resources increase. This further facilitates a spatial structure where dominance, exclusion and even sympatry are all observed (figure 3*i*,*j*).

Argyrodes spiders present an intriguing example that parallels our findings. Approximately 50 species of orb-web spiders, such as *Metepeira incrassata*, are considered social and dwell in group webs ([52]; see also [53]). The webs structurally support one another, thereby reducing maintenance, and increasing prey-capture efficiency of the web complex. These webs are also home for several species of *Argyrodes* (e.g. *A. elevatus*, *A. globbussus*; also see Peng *et al.* [54] for potential mutualism with *A. fissifrons*) who survive as inquiline kleptoparasites and occasional facultative predators. *Argyrodes* spp. have forgone the production of their own webs and instead feed on captured prey before or even during host feeding. Their activities can be sufficient to motivate hosts to abandon a web altogether, echoing the host displacement in our model. It is unclear how the kleptoparasite initially locates a host web, but it has been hypothesized that they may actively trail a silk train left by the vacating host spider of a previously parasitized web [55], which would clearly constitute adaptive movement of individuals. Thrips offer a similar example where kleptoparasites take over insect-induced galls that provide shelter and food for their inhabitants [8]. Notably, the incidence of parasitism is greater in more social lineages.

In experiments controlling the distribution of food (suet), blackbirds (*Turdus merula L.*) engaged in theft only when food was clumped but not when it was spread out [42], suggesting a conditional trade off in behavioural strategies. Datta *et al.* [32] experimentally demonstrated that cooperation could be maintained via range expansion in yeast travelling across a series of wells. An obligate parasite strain of yeast lacked the ability to catalyse the conversion of sucrose and relied upon other strains to obtain glucose and fructose. The expansion velocity of co-operators was greater than that of the defector strain, probably due to an advantage of co-operators at low concentrations. Defectors were delayed until host densities in adjoining wells attained sufficient size. Within our model, this exact result is unlikely to be present as co-operators are disinclined to expand into novel territory, yet it does match the observed behaviour of selfish hosts (figure 6*b*,*c*). In our community simulations, the speed of the selfish expansion wave exceeded that of the trailing parasites. The ability to outrun the parasite is limited in a heterogeneous environment as hosts ultimately encounter marginal regions and stall their migration [2].

Cosner & Nevai [56] modelled kleptoparastism in a diffusive producer–scrounger model, roughly corresponding to the selfish host–parasite dynamic discussed here. They found similar resource dependence in the spatial structure of the community and that slow movement aided the persistence of each group. During intra-guild predation, predators occupied areas of high resource while prey were marginalized to poorer locations, but refuges did aid in the persistence of the prey species [7]. Finally in a model of preytaxis, the sensitivity of the scrounger to variations in host density could push spatio-temporal patterning [57]. Sensitivity to environmental variations (parameters *k*_*i*_) was equal to or slower than other processses in many of our simulations. This may have obscured the emergence of spatio-temporal patterning, so these other results suggest future directions for the model presented here.

The functional form of fitness is the first contrasted feature under this modelling framework in which the natural ideal distribution is marked by the mutual exclusion of two populations (selfish versus cooperative). Rowell [3] studied the competition between two selfish populations that exhibited various performance asymmetries. Differences in resource collection drove the system to parapatry, culminating in the elimination of the ‘clumsier’ population which clustered about resource peaks. By contrast, interference dominance marginalized weaker individuals, but with an intermediate zone of sympatry. Secondary variations in interference strength could preserve the less fit population via either mutual exclusion (strong interspecific interference) or sympatry (weak interspecific interference). Suárez [58] and Namba [59], similarly reported coexistence for diffusive models under highly aggressive interactions between populations and when interspecific pressures were less than intraspecific ones, respectively. In the model here, reducing interspecific interference promotes sympatry only for limited areas of the environment. Resource-rich areas enforced mutual exclusion among host competitors. Under parasitism, the mismatch of consumer to resource (or parasite to prey) seen at each ecological link yields distributions comparable to performance trade-offs between resource collection (parameter *h* in our model) and interference dominance (*a*_*ij*_) in Rowell [3]. Because hosts possess no effective deterrent to parasites, the kleptoparasites take up the ‘clumsy but strong’ persona while the hosts—as the ‘weak but skilled’ population—cleave to a refuge in marginal areas. Unlike in the performance trade-off, parasites cannot hold exclusive control over resource peaks and either must share it with a productive host or destabilize the local dynamics such that neither population occupies the area. Requeio & Camacho [60] similarly discovered that marginal value refuges could protect co-operators from defectors in a simulation study of network games.

Every regional community conforms to an underlying spatial structure of its sub-populations. The selfish host population attains its natural range through its capacity for colonization, while the kleptoparasite follows behind without hindrance (figure 3*a*–*d*). The development of other communities requires favourable historical distributions and locations of introductions. Physical barriers, established heterospecifics and resource deserts could restrict a species' range (figure 3*i*,*j*). In our example of sequential introductions (figure 6), selfish newcomers had a natural aggregation site at the resource peak, but pre-existing co-operators in low-resource refuges prevented access to the centre if they arose in the margin. As shown, though, favourable positions could lead to a widespread expansion in the interior of the environment and bridge the distinct areas of habitation. This fact is a precautionary warning about the risk of hidden physical or demographic corridors connecting locations which could facilitate rapid invasions and epidemics after barriers are breached in a seemingly stable community.

The resource dependence of coexistence and instability leads to important conclusions for climate change. Different aspects of our model could be susceptible to environmental changes, but the resource curve is the simplest proxy of environmental shifts. A gradual increase in temperature can result in an increase in resources, or more generally the energetics of the ecosystem. This in turn destabilizes areas with previously manageable levels of parasitism and forces cohabitating niche competitors into antagonistic exclusion. When parasitized, even selfish populations decline as resources become more abundant. This runs counter to a ‘rising tide’ argument that all consumers might benefit from an increase of resources. If resources instead decline, parasites would have a diminished presence in the community, to the benefit of both hosts, but co-operators would also contract their home range relative to selfish individuals.

Similar social implications pertain to the care of dependents (juvenile or brood care) or non-productive individuals within a group. Supported individuals include the elderly or senescent individuals but also those who fulfill caste-roles but do not contribute directly to the accumulation of resources, e.g. those engaged in the defence of group territory or social/physical maintenance of the group. This could drive a co-evolution of transfer rates and mechanisms to regulate group size or social composition to mitigate the overtaxation of producers and prevent destabilization. One epidemiological interpretation of our model is to consider kleptoparasites as carriers of a juvenile-age disease that renders infected individuals infertile, with cross-generational transmission between infected adults and new offspring or juveniles. The co-evolution of disease traits and social behaviours in response to the spread of the infection becomes central [61].

Our findings suggest a number of promising options for continued research using ideally motivated populations. A number of functional alternatives describe parasitism and predation, and some predatory activities, e.g. harvesting and trapping, may be non-observable to prey (J.T.R. 2016, unpublished manuscript). Different hunting and avoidance rules invite contrasts in range limits, refuges and distributions. Second, although co-operators could exclude selfish competitors and maintain refuges from parasites in low-value areas, the origin of cooperation remains unidentified. We consider three candidate hypotheses appropriate for further examination within this framework: (i) mutation between selfishness and cooperation; (ii) stepwise replacement via intermediate trait groups; and finally, (iii) conditional switching of behaviour with subsequent canalization. The consequences of climatic variation (spatial, demographic, epidemiological, etc.) through changes in resources and other model features offer another direction. In addition to the resource-mediated effects that we have observed, climate changes such as temperature could affect aggression levels or shift the location of clinal tolerances, thereby forcing new alignments within the wider community. Finally, the disruption caused by exploitation, the sudden contact between different groups, and the loss of coexistence between selfish and cooperative groups as resources increase have socio-economic implications that warrant exploration in their own context.

## Appendix A

This appendix establishes several conditions and relationships concerning kleptoparasitic invasions.

**A.1. Minimum host density**

The local kleptoparasite density is governed by the equation

A 1 $$\frac{du_{3}}{dt}=r_{3}u_{3}\left(\theta_{i}u_{i}{\tilde{f}}_{i}-\frac{\mu_{3}}{r_{3}}\right),$$

where ${\tilde{f}}_{i}$ is the natural fitness function of host *i*. Parasites will invade if the growth rate is positive, which occurs when

A 2 $$\theta_{i}u_{i}{\tilde{f}}_{i}>\frac{\mu_{3}}{r_{3}}.$$

If the host is already at equilibrium prior to the introduction of the parasite, then ${\tilde{f}}_{i}=\mu_{i}/r_{i}$ [2], and the condition becomes

A 3 $$\theta_{i}u_{i}\frac{\mu_{i}}{r_{i}}>\frac{\mu_{3}}{r_{3}}.$$

Isolating *u*_*i*_ gives condition (3.1) in the main text.

**A.2. Linear constraint on host and parasite densities**

If a host and kleptoparasitic population coexist at a local dynamic equilibrium (${\tilde{u}}_{i},{\tilde{u}}_{3})$, then the densities must simultaneously satisfy

A 4 $$(1-\theta_{i}{\tilde{u}}_{3}){\tilde{f}}_{i}({\tilde{u}}_{i})-\frac{\mu_{i}}{r_{i}}=0$$

and

A 5 $$\theta_{i}{\tilde{u}}_{i}{\tilde{f}}_{i}({\tilde{u}}_{i})-\frac{\mu_{3}}{r_{3}}=0.$$

By first moving the ratios of mortality to metabolic efficiency to the right hand side of the equations, we can then divide one equation by the other to obtain

A 6 $$\frac{\left(1-\theta_{i}{\tilde{u}}_{3}){\tilde{f}}_{i}({\tilde{u}}_{i}\right)}{\theta_{i}{\tilde{u}}_{i}{\tilde{f}}_{i}({\tilde{u}}_{i})}=\frac{\left(1-\theta_{i}{\tilde{u}}_{3}\right)}{\theta_{i}{\tilde{u}}_{i}}=\frac{\mu_{i}/r_{i}}{\mu_{3}/r_{3}}.$$

This further simplifies to

A 7 $$(1-\theta_{i}{\tilde{u}}_{3})=\theta_{i}{\tilde{u}}_{i}\left(\frac{\mu_{i}}{r_{i}}\right)\left(\frac{r_{3}}{\mu_{3}}\right).$$

Isolating ${\tilde{u}}_{3}$ yields relationship (3.2) in the main text.

**A.3. Stability of coexistence in non-spatial models**

Suppose that there is a local coexistence state for a single host and the kleptoparasite, (${\tilde{u}}_{i},{\tilde{u}}_{3})$. The Jacobian matrix for this system is

A 8 $$\left[\begin{array}{cc} r_{i}\left[(1-\theta_{i}{\tilde{u}}_{3}){\tilde{f}}_{i}({\tilde{u}}_{i})-\frac{\mu_{i}}{r_{i}}\right]+r_{i}{\tilde{u}}_{i}(1-\theta_{i}{\tilde{u}}_{3}){\tilde{f}}_{i}^{\prime }({\tilde{u}}_{i}) & -\theta_{i}r_{i}{\tilde{u}}_{i}{\tilde{f}}_{i}({\tilde{u}}_{i})\\ r_{3}{\tilde{u}}_{3}\theta_{i}[{\tilde{f}}_{i}({\tilde{u}}_{i})+{\tilde{u}}_{i}{\tilde{f}}_{i}^{\prime }({\tilde{u}}_{i})] & r_{3}\theta_{i}\left[{\tilde{u}}_{i}{\tilde{f}}_{i}({\tilde{u}}_{i})-\frac{\mu_{3}}{r_{3}}\right] \end{array}\right],$$

which can be further reduced to

A 9 $$\left[\begin{array}{cc} r_{i}{\tilde{u}}_{i}(1-\theta {\tilde{u}}_{3}){\tilde{f}}_{i}^{\prime }({\tilde{u}}_{i}) & -\frac{r_{i}\mu_{3}}{r_{3}}\\ r_{3}\tilde{u_{3}}\theta_{i}({\tilde{f}}_{i}({\tilde{u}}_{i})+{\tilde{u}}_{i}{\tilde{f}}_{i}^{\prime }({\tilde{u}}_{i})) & 0 \end{array}\right].$$

If the host is selfish, ${\tilde{f}}_{1}^{\prime }=-a_{11}R/{\left(a_{11}{\tilde{u}}_{1}+h\right)}^{2}<0$, and ${\tilde{f}}_{1}+u_{1}{\tilde{f}}_{1}^{\prime }=Rh/{\left(a_{11}{\tilde{u}}_{1}+h\right)}^{2}>0$. Consequently, the Jacobian always has a negative trace and positive determinant, i.e. the equilibrium is always locally stable.

If the host is cooperative, however, ${\tilde{f}}_{2}^{\prime }=R[\alpha^{2}-a_{22}u_{2}^{2}]/{\left(a_{22}u_{2}^{2}+\alpha^{2}\right)}^{2}$, which is of variable sign, while ${\tilde{f}}_{2}+{\tilde{u}}_{2}{\tilde{f}}_{2}^{\prime }=2R{\tilde{u}}_{2}\alpha^{2}/{\left(a_{22}u_{2}^{2}+\alpha^{2}\right)}^{2}>0$. The determinant is thus always positive, so stability rests solely on the sign of the trace, and more specifically ${\tilde{f}}^{\prime }$.

Setting the derivative ${\tilde{f}}_{2}^{\prime }=0$, one finds that the sign change occurs when ${\tilde{u}}_{2}=\alpha /\sqrt{a_{22}}$; however, solving directly for ${\tilde{u}}_{2}$ via the kleptoparasite's differential equation, we also know that the equilibrium value is ${\tilde{u}}_{2}=\alpha /\sqrt{\theta_{2}Rr_{3}/\mu_{3}-a_{22}}$. Equating the two solution forms, the transition from stable to unstable spirals occurs at the resource level

A 10 $$R=\frac{2a_{22}(\mu_{3}/r_{3})}{\theta_{2}}.$$

**A.4. Local host interactions**

Consider the local, non-spatial dynamics for a community consisting only of hosts. There are four possible types of equilbria in this model: the trivial solution (0,0), the semi-trivial solutions $\left(u_{1}^{*},0\right)$ and $\left(0,u_{2}^{*}\right)$, and the coexistence state $\left(u_{1}^{*},u_{2}^{*}\right)$.

It is previously established that the trivial solution is unstable if *R*>*R*_via_ for the selfish host. The semi-trivial solution $\left(u_{1}^{*},0\right)$ is locally stable as a consequence of the Allee effect on co-operator fitness. The equilibrium has eigenvalues $\lambda_{2}=r_{1}(\partial f_{1}/\partial u_{1})u_{1}^{*}$ and *λ*_1_=−*μ*_2_, both of which are negative.

The semi-trivial solution (0,*u**_2_) has eigenvalues $\lambda_{1}=r_{1}f_{1}(0,u_{2}^{*})-\mu_{1}$ and $\lambda_{2}=r_{2}(\partial f_{2}/\partial u_{2})u_{2}^{*}$. Assuming that we are considering the upper solution for co-operator density, *λ*_2_<0. The solution is stable to selfish invasions if

A 11 $$\mu_{1}>r_{1}f_{1}(0,u_{2}^{*}),$$

otherwise, the equilibrium is unstable, which leads to (3.5) in the main text.

For possible coexistence states, the Jacobian matrix is

A 12 $$\left[\begin{array}{cc} r_{1}\frac{\partial f_{1}}{\partial u_{1}}u_{1}^{*} & r_{1}\frac{\partial f_{1}}{\partial u_{2}}u_{1}^{*}\\ r_{2}\frac{\partial f_{2}}{\partial u_{1}}u_{2}^{*} & r_{2}\frac{\partial f_{2}}{\partial u_{2}}u_{2}^{*} \end{array}\right].$$

The determinant of the matrix above is Δ=*r*_1_*r*_2_*u**_1_*u**_2_((∂*f*_1_/∂*u*_1_)(∂*f*_2_/∂*u*_2_)−(∂*f*_2_/∂*u*_1_)(∂*f*_1_/∂*u*_2_)). Using the fitness functions of this model,

A 13 $$\Delta =\frac{r_{1}r_{2}u_{1}^{*}u_{2}^{*}(-a_{11}(a_{11}u_{1}^{*}+a_{12}{u_{2}^{*}}^{2}+h_{1})+2(a_{11}a_{22}-a_{12}a_{21}){u_{2}^{*}}^{2})}{{\left(a_{11}u_{1}^{*}+a_{12}{u_{2}^{*}}^{2}+h_{1}\right)}^{2}{\left(a_{21}u_{1}^{*}+a_{22}{u_{2}^{*}}^{2}+\alpha_{2}^{2}\right)}^{2}}.$$

The point is a saddle if

A 14 $$a_{11}(a_{11}u_{1}^{*}+a_{12}{u_{2}^{*}}^{2}+h_{1})>2(a_{11}a_{22}-a_{12}a_{21}){u_{2}^{*}}^{2},$$

which is always true under balanced antagonism (*a*_11_*a*_22_=*a*_12_*a*_21_).

**A.5. Numerical simulations**

The numerical simulations and plots were generated with MATLAB R2013a on a Dell Precision T3600 Workstation running Windows 7. Trajectories for non-spatial models were obtained by a simple forward-time step Euler method. Nullclines and equilibria were obtained analytically.

For spatially dependent models, time advancement was again handled with a forward time step with change step Δ*t*=0.0002. Depending on the simulation, the programs were run for 50 000, 100 000 or 125 000 iterations, corresponding to end times of 10, 20 and 25 time units, respectively. The spatial environment was discretized into patches of width Δ*x*=0.05. Spatial derivatives were obtained using MATLAB's native gradient function which applies a form of central-difference method. Adjustments were applied at jumps where populated locations neighboured 0-fitness locations as the gradient function artificially induced movement flux at these sites.
